# Control of the induction of type I interferon by Peste des petits ruminants virus

**DOI:** 10.1371/journal.pone.0177300

**Published:** 2017-05-05

**Authors:** Beatriz Sanz Bernardo, Stephen Goodbourn, Michael D. Baron

**Affiliations:** 1 The Pirbright Institute, Pirbright, Surrey, United Kingdom; 2 Institute for Infection and Immunity, St George’s, University of London, London, United Kingdom; University of Hong Kong, HONG KONG

## Abstract

*Peste des petits ruminants virus* (PPRV) is a morbillivirus that produces clinical disease in goats and sheep. We have studied the induction of interferon-β (IFN-β) following infection of cultured cells with wild-type and vaccine strains of PPRV, and the effects of such infection with PPRV on the induction of IFN-β through both MDA-5 and RIG-I mediated pathways. Using both reporter assays and direct measurement of IFN-β mRNA, we have found that PPRV infection induces IFN-β only weakly and transiently, and the virus can actively block the induction of IFN-β. We have also generated mutant PPRV that lack expression of either of the viral accessory proteins (V&C) to characterize the role of these proteins in IFN-β induction during virus infection. Both PPRV_ΔV and PPRV_ΔC were defective in growth in cell culture, although in different ways. While the PPRV V protein bound to MDA-5 and, to a lesser extent, RIG-I, and over-expression of the V protein inhibited both IFN-β induction pathways, PPRV lacking V protein expression can still block IFN-β induction. In contrast, PPRV C bound to neither MDA-5 nor RIG-I, but PPRV lacking C protein expression lost the ability to block both MDA-5 and RIG-I mediated activation of IFN-β. These results shed new light on the inhibition of the induction of IFN-β by PPRV.

## Introduction

Peste des petits ruminants (PPR) is a viral disease of sheep, goats and related wild animals. It is caused by the morbillivirus *Peste des petits ruminants virus* (PPRV), which is closely related to *Rinderpest virus* (RPV), *Canine distemper virus* (CDV) and *Measles virus* (MeV). PPRV is widely distributed in the African and Asian continents (see [1] for a recent review), and it can be classified in four genetic lineages based on the sequence of short segments of either the F or the N genes [2–4]. However, all four lineages share the same serotype, and a single vaccine strain, based on a Nigerian isolate, has provided complete protection against disease from West Africa to China. The disease is characterized by conjunctivitis, rhinitis, stomatitis, pneumonia and enteritis, and also immune-suppression [5–10]. The severity of the clinical signs, from mild to severe, varies with the virus isolate and with the host [10–13].

Morbilliviruses are negative-sense single-stranded RNA viruses of the family *Paramyxoviridae*. They have six genes and encode eight proteins, with three separate proteins encoded by the P gene: P, C and V [14, 15]. The P protein is produced from mRNAs that are a direct transcript of the P gene, while V is generated by co-transcriptional editing of the mRNA transcript, inserting a single G residue about half way along the coding sequence, thereby switching the reading frame [16, 17]; the V and P proteins therefore differ only in their carboxy termini. Viruses of the other genera of *Paramyxoviridae*, such as *Sendai virus*, *Newcastle disease virus* and *Parainfluenza virus type 5* (PIV5), also make a V protein. The morbillivirus C protein is generated from an internal open reading frame (ORF) in all P gene transcripts; respiroviruses, such as *Sendai virus* (SeV), also produce C proteins, and in a similar way, but there is no sequence similarity between the C proteins of the two genera. Rubulaviruses do not make a C protein. There is extensive evidence that both V and C proteins are involved in controlling the host’s interferon (IFN) responses [18–20].

The initial stage in the type I IFN response is the production of IFN-β, which usually occurs soon after cell infection, and requires various transcription factors to bind to the regulatory domains of the IFN-β promoter [21–27]. Transcription factor activation occurs when the infected cells recognize an invading pathogen, through detection of specific pathogen associated molecular patterns (PAMPs) by cellular pattern recognition receptors (PRR). Retinoic acid-inducible gene I (RIG-I)-like receptors (RLR) are a type of PRR which detect intracellular pathogens by the specific RNA forms that they make. This family of PRRs includes the proteins RIG-I, melanoma differentiation-associated protein 5 (MDA-5) and laboratory of genetics and physiology 2 (LGP2). All three RLRs have a DExH/D box RNA helicase domain and a specific carboxy-terminal domain; in addition, MDA-5 and RIG-I have two tandem caspase recruitment domains (CARDs) at their amino-terminus which are involved in downstream signalling leading to activation of the IFN-β promoter (reviewed in [28]). The role of LGP2, which lacks a CARD, has been reported as the facilitation of MDA-5 activation, and the repression of RIG-I [29–31]. It has also been suggested that PAMP detection by both MDA-5 and RIG-I may be facilitated by LGP2 [32].

The paramyxovirus V proteins bind to MDA-5 and block the MDA-5-mediated induction of the IFN-β promoter [33–35]. Additionally, the V protein of MeV has been shown to bind to phosphoprotein phosphatase 1 (PP1), preventing the dephosphorylation of MDA-5 [36] that is required for MDA-5 downstream signalling. The PIV5 V protein does not bind to RIG-I nor directly inhibit its activity [34], but has been found to bind to LGP2 [37, 38], and this interaction has been proposed to mediate inhibition of the IFN-β promoter activation when this occurs through RIG-I [38]. It has also been reported that, although LGP2 enhances the MDA-5-mediated activation of the IFN-β promoter, the PIV5 V protein can still block MDA-5 when bound to LGP2 [38, 39]. Therefore, the paramyxovirus V protein appears to inhibit the induction of IFN-β following either MDA-5 or RIG-I activation.

The role of the paramyxovirus C proteins in the induction of IFN-β is unclear. The morbillivirus C protein is involved in the replication of viral genome, in the regulation of RNA synthesis [40–42] and in the translation of viral proteins [43], activities which could lead to evasion of the activation of the IFN-β promoter. Viruses engineered to not produce C have been shown to synthesize more double-stranded RNA (dsRNA) during infection, a PAMP which would lead to activation of transcription from the IFN-β promoter through protein kinase R (PKR) [44, 45] as well as through MDA-5, whereas dsRNA is not produced by wild type viruses [46–48]. A direct effect of the C protein in blocking IFN-β transcription has also been suggested [49].

We have investigated the effect of PPRV infection on the induction of IFN-β, and the role of its accessory proteins (V and C) on the MDA-5 and RIG-I signalling pathways. We have also studied the role of V and C during infection with PPRV by generating mutant viruses which express either V or C, but not both.

## Results

### PPRV infection does not lead to induction of IFN-β

**Fig 1:**
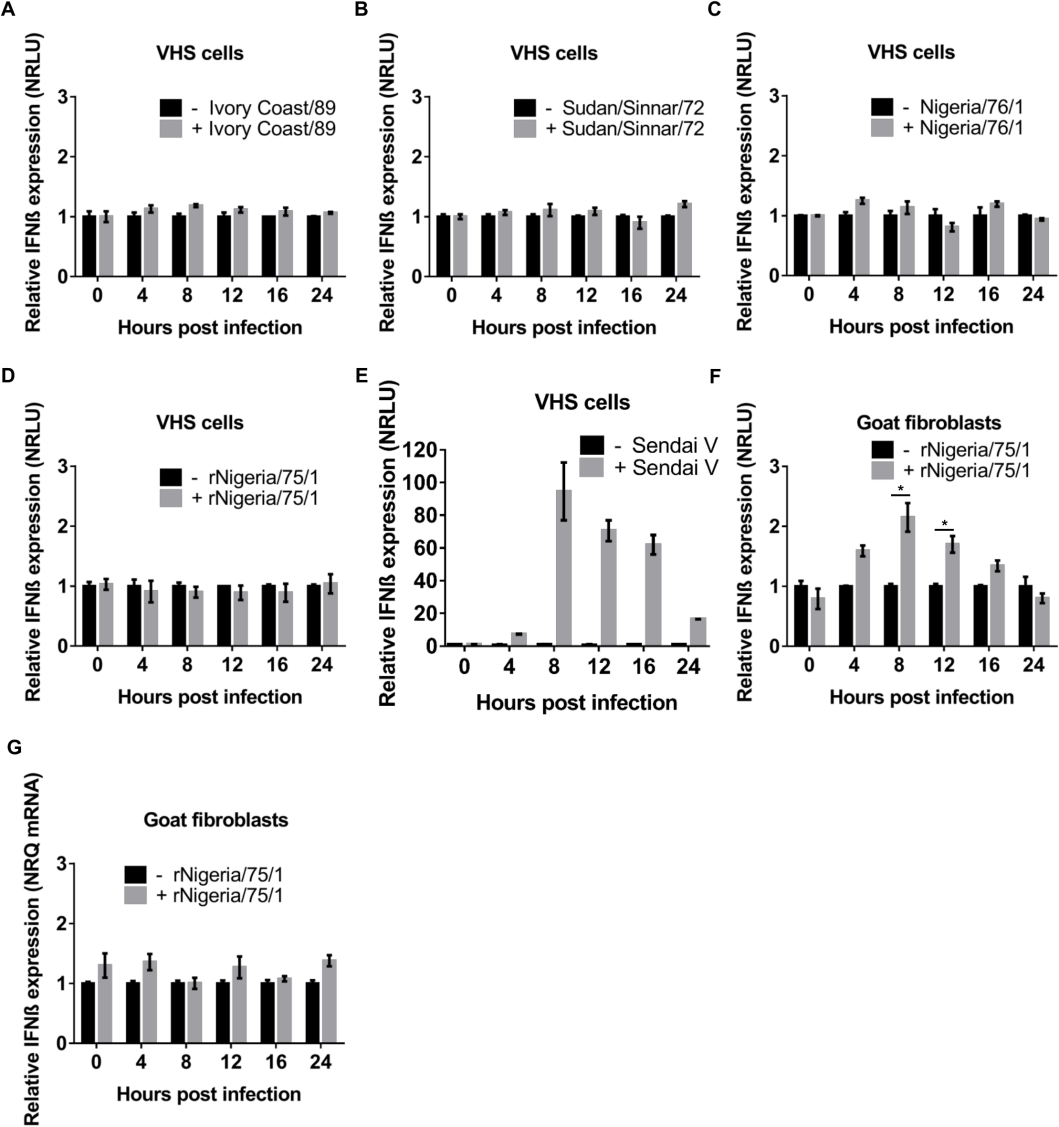
IFN-β induction during the infection with different strains of PPRV. VHS cells (A-E) or G4 cells (F) were transfected with pIFN-β-luc (350 ng) and pJAT-lacZ (200 ng). At least 18 hours post transfection, cells were infected with (A-D) the indicated strain of PPRV at a MOI = 1 TCID_50_/cell or (E) with the Cantell strain of Sendai virus (50 HA units/well); in each case parallel samples were left uninfected. Duplicate samples were taken at each time point and cell extracts were prepared and assayed for luciferase and β-galactosidase activity as described in Methods. The luciferase reading was expressed relative to the β-galactosidase activity (Relative Light Units (RLU)). Induction of the IFN-β promoter was expressed as the ratio of RLUs in infected cells relative to that in uninfected cells (set as 1 for each time point). (G) G4 cells were infected with PPRV as in (F) and triplicate samples of cells harvested at the indicated times and RNA extracted. IFN-β mRNA was measured by RT-qPCR as described in Methods and normalized by setting the value for uninfected cells to 1. Error bars represent standard error of the mean (SEM). The ANOVA test and Tukey pairwise comparisons test were used to compare differences between the means (* = p < 0.05).

Initially, we tested whether infection with PPRV stimulated the production of IFN-β, using PPRV-infected Vero-human-SLAM (VHS) cells which had previously been transfected with a reporter plasmid expressing luciferase under the control of the IFN-β promoter and a transfection control plasmid constitutively expressing β-galactosidase. Vero cells do not produce a functional type I IFN [50–52], and have therefore been extensively used to study the activation of the IFN-β promoter; the presence of the SLAM (signalling lymphocytic activation molecule) protein makes these cells highly susceptible to morbilliviruses [53], including PPRV. IFN-β induction was measured as the relative activity of luciferase and β-galactosidase. We infected transfected VHS cells with a series of field viruses representing three of the lineages of PPRV (Ivory Coast/89, Sudan/Sinnar/72 and Nigeria/76/1) and measured the activation of the IFN-β promoter over the following 24 hours. We confirmed by immunofluorescence (IF) that > 95% of the cells were infected with PPRV (not shown). During the first 24 hours post infection (hpi), the IFN-β promoter was not activated in cells infected by any of the wild type PPRV strains (Fig 1A–1C). We also tested the effect of infection with the PPRV vaccine strain (Nigeria/75/1) [54], using a recombinant version [55], which we also used in later experiments to introduce mutations into PPRV; we observed that this virus also failed to activate the IFN-β promoter (Fig 1D). In contrast, in control studies where the cells were infected with a preparation of Sendai virus known to induce IFN-β (SeV-DI), a strong activation of expression from the reporter plasmid was seen (Fig 1E) [56].

To study the effect of PPRV infection in cells derived from one of the natural hosts of the virus, we carried out the same experiment in primary goat skin fibroblasts (G4 cells). These cells, unlike VHS cells, have a functional type I IFN gene and therefore have a positive feedback loop following the initial expression of IFN-β [57]. When we transfected the reporter plasmids into the G4 cells and infected them with the recombinant PPRV, we consistently observed a small and transient activation of the IFN-β promoter, decreasing to almost background levels by 16 hpi (Fig 1F). Since the primary goat fibroblasts can synthesize IFN-β, we attempted to confirm this finding by measuring the amount of IFN-β mRNA by RT-qPCR, but no significant change in the levels of IFN-β mRNA was observed following the infection of these cells with PPRV (Fig 1G). It is possible that the direct measurement of IFN-β mRNA is less sensitive than the enzyme-based assay, or that the cellular (chromatin) promoter is less sensitive to activation than that on the plasmid.

### PPRV infection actively blocks MDA-5 and RIG-I mediated induction of IFN-β

**Fig 2:**
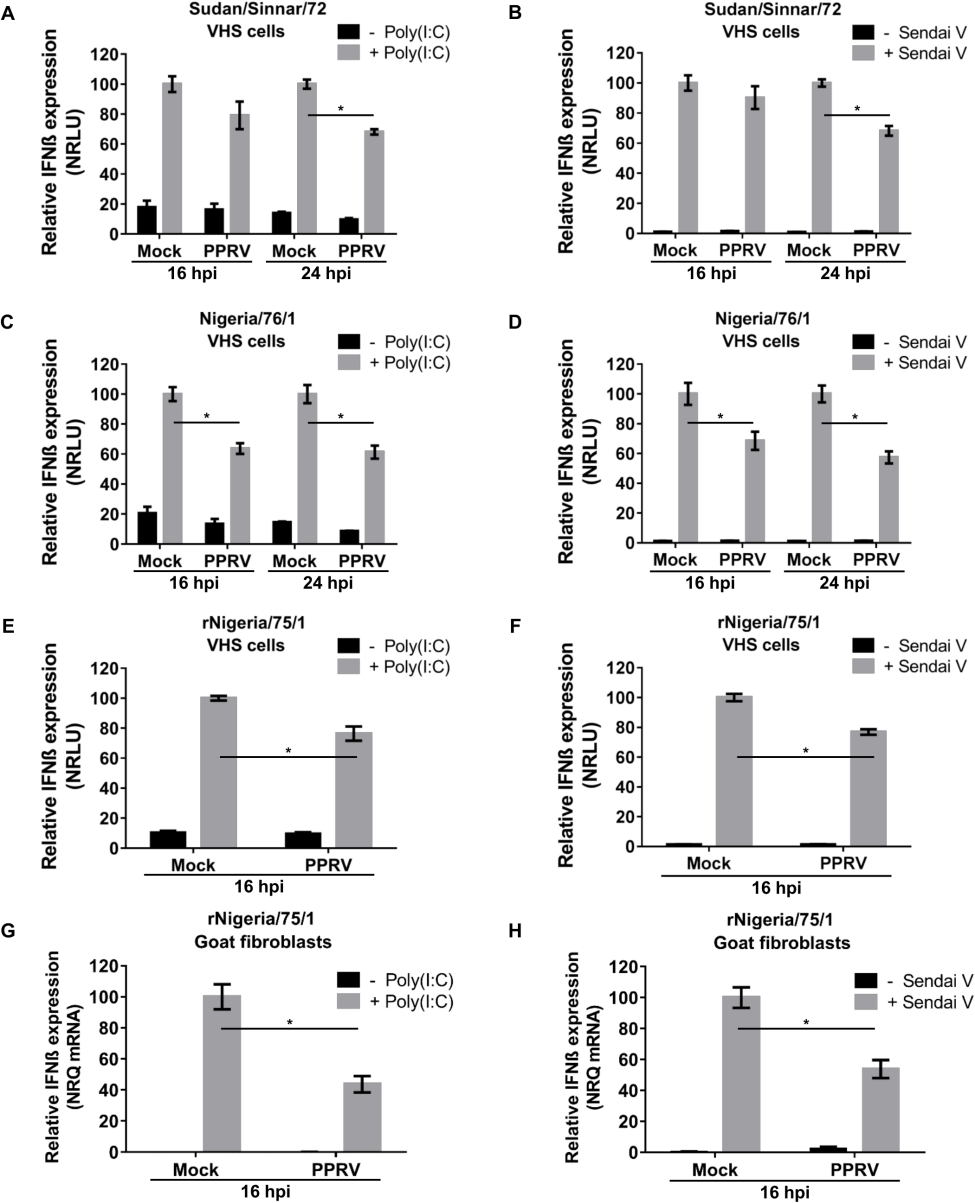
PPRV actively blocks MDA-5 and RIG-I mediated induction of the IFN-β promoter. VHS cells (A-F) were transfected with pIFN-β-luc (350 ng) and pJAT-lacZ (200 ng). Additionally, for the poly(I:C)-mediated induction of IFN-β (A, C, E), 100 ng of MDA-5 plasmid was added to the transfection mix. Cells were infected with the indicated strain of PPRV at MOI = 3 for the indicated time, or left uninfected, before transfection of poly(I:C) (A, C, E, G) or infection with SeV-DI (B, D, F, H). Cell extracts were prepared from triplicate samples and were assayed for luciferase and β-galactosidase activity (A-F) or for IFN-β mRNA (G, H) as described in Methods. Results were normalised by setting the RLU, or the level of IFN-ß mRNA, in treated uninfected cells as 100. Error bars represent the SEM. The ANOVA test and Tukey pairwise comparisons test were used to compare differences between the means (* = p < 0.05).

These experiments suggested that either PPRV is avoiding induction of IFN-β or that PPRV infection is preventing the induction of IFN-β by actively supressing the activation of the promoter. We therefore studied the effect of PPRV infection on activation of the IFN-β promoter by PAMPs. We used transfected poly(I:C) to activate MDA-5-mediated IFN-β induction and SeV-DI to activate RIG-I-mediated induction. We confirmed that these reagents specifically activated through their respective pathways using plasmids encoding dominant negative forms of MDA-5 and RIG-I [35] (S1 Fig). We studied whether field isolates of PPRV (Sudan/Sinnar/72, Nigeria/76/1) were able to actively block the induction of IFN-β. We transfected VHS cells with the reporter plasmids, infected them with PPRV (multiplicity of infection (MOI) = 3) and then, at 16 hpi or 24 hpi, treated them with either poly(I:C) or SeV-DI. We confirmed by immunofluorescence that PPRV-infected cells could still be infected with SeV (S2 Fig). Incomplete but reproducible suppression of IFN-β induction by PPRV/Sudan/Sinnar/72 was observed when the stimulus was applied at 24 hpi, but not at 16 hpi (Fig 2A and 2B). In VHS cells infected with PPRV/Nigeria/76/1, suppression of the induced expression from the IFN-β promoter could be observed already at 16 hpi, as well as at 24 hpi (Fig 2C and 2D). The vaccine strain of PPRV also appeared to actively suppress IFN-β induction, in both VHS cells using the luciferase reporter assay (Fig 2E and 2F), and in G4 cells using the direct measurement of IFN-β mRNA (Fig 2G and 2H).

### The V protein of PPRV actively blocks IFN-β induction by poly(I:C) and binds to MDA-5

**Fig 3:**
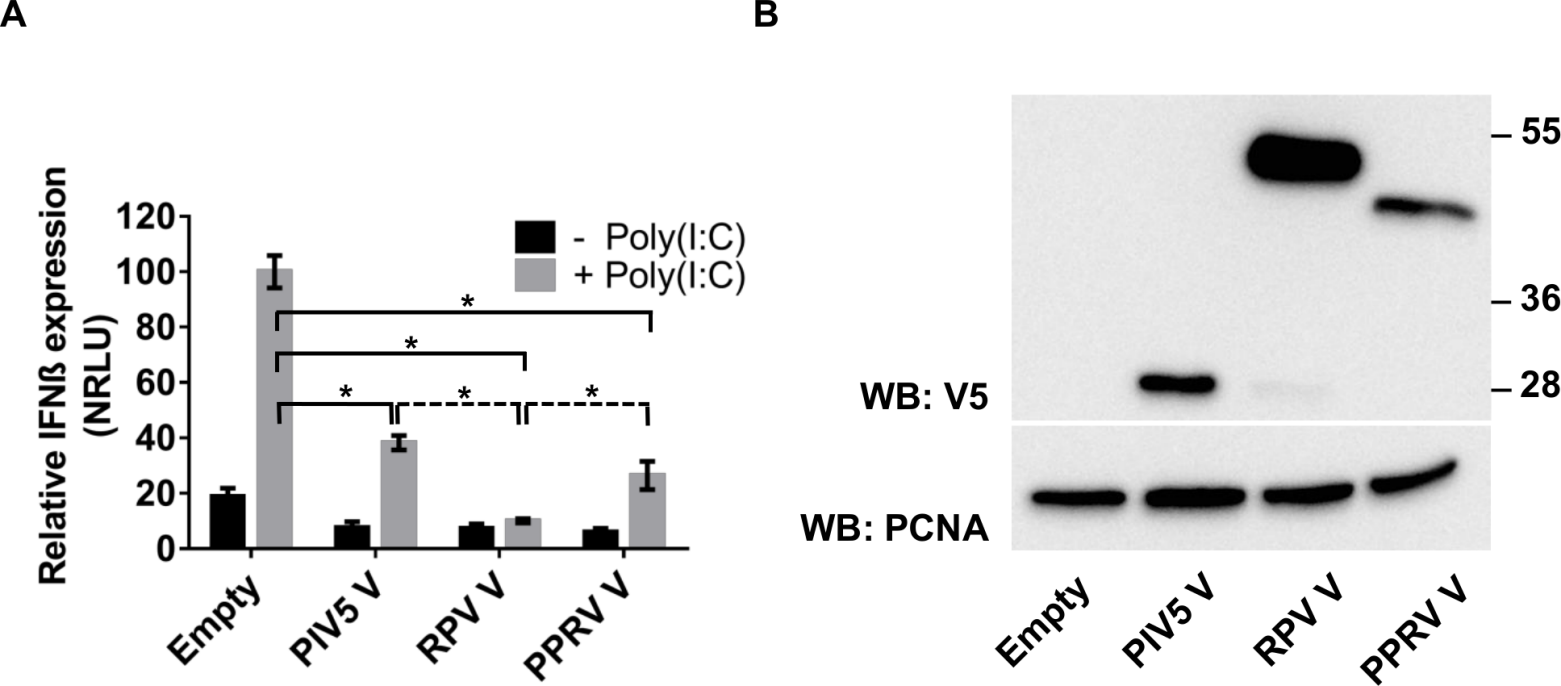
The V protein of PPRV blocks the induction of IFN-β by intracellular poly(I:C). (A) VHS cells were transfected with pIFN-β-luc (350 ng), pJAT-lacZ (200 ng), pEF-MDA-5 (100 ng) and an empty plasmid (500 ng) or expression plasmid encoding PIV5 V (500 ng), RPV V (300 ng) or PPRV V (500 ng). The total amount of DNA was kept constant in all samples by adding empty plasmid as required. At least twenty four hours post transfection, cells were transfected with 2 μg of poly(I:C) or left untreated; 7 hours later the cells were lysed and the cells extracts were assayed for luciferase and β-galactosidase activities. Samples were normalised by setting RLUs in poly(I:C) transfected cells without V protein to 100. Error bars represent the standard error of the mean. ANOVA and Tukey pairwise comparison test were used to determine the statistical significance of differences in means (* = p < 0.05). (B) Cell extracts from parallel transfected wells were run on 10% SDS-PAGE gels and Western blotted with anti-V5 or anti-PCNA (loading control).

Similar to other paramyxoviruses, PPRV encodes a V protein that has a cysteine-rich C-terminal domain closely resembling that which has been reported to be an MDA-5 antagonist in other paramyxoviruses, actively blocking the activation of the IFN-β promoter through the MDA-5-mediated signalling pathway [33–35]. We tested if the expression *in trans* of the PPRV V protein was also able to block the MDA-5-mediated activation of the IFN-β promoter, comparing it to the V protein of PIV5 (the paramyxovirus that has been the model to study the role of the V protein) and the V protein of another morbillivirus, RPV, which has previously been shown to affect host IFN signalling [58–60]. We transfected VHS cells with the reporter plasmids and plasmids encoding the selected V proteins and induced the activation of the MDA-5 signalling cascade by transfection of poly(I:C). All three V proteins actively blocked this pathway (Fig 3).

**Fig 4:**
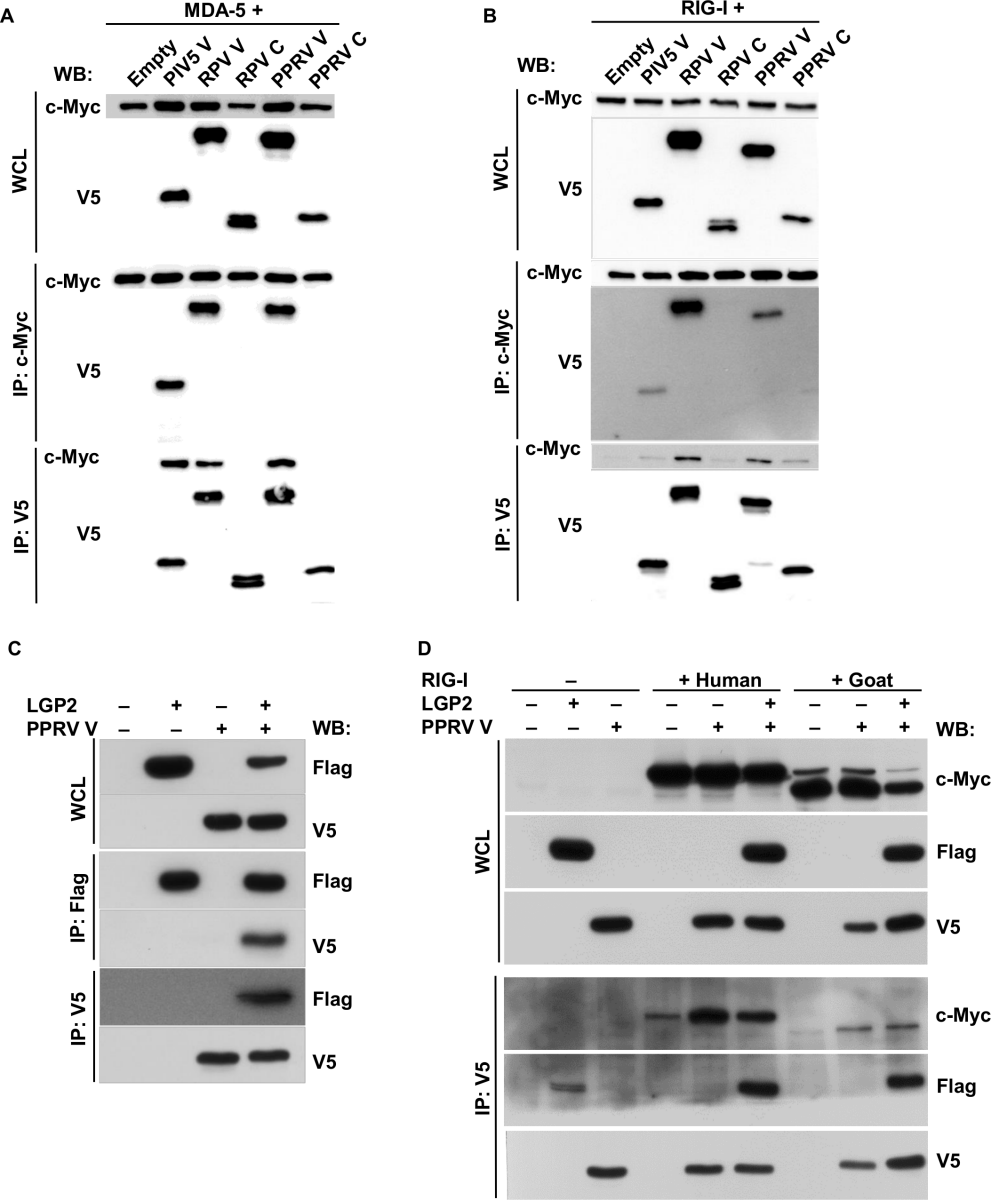
The V protein of PPRV binds to MDA-5, RIG-I and LGP2. HEK-293FT cells were transfected with plasmids encoding (A) c-Myc-MDA-5 (1400 ng) and either an empty plasmid (1400 ng) or a plasmid encoding one of the viral accessory proteins PIV5 V (1400 ng), RPV V (100 ng), RPV C (400 ng), PPRV V (300 ng) or PPRV C (100 ng); (B) c-Myc-RIG-I (1400 ng) and the same set of empty and expression plasmids as in (A); (C) an expression plasmid encoding Flag-goat LGP2 (500 ng) and an empty plasmid (300 ng) or an expression plasmid encoding the PPRV V protein (300 ng); (D) expression plasmids encoding c-Myc-human RIG-I (1200 ng), c-Myc-goat RIG-I (2600 ng), Flag-goat LGP2 (500 ng), PPRV V (200 ng) and/or empty plasmid as indicated. The total amount of DNA was kept constant in all samples by adding empty plasmid as required. (A-D) Forty eight hours post transfection, cells were lysed and cell extracts were immune-extracted using antibodies against c-Myc and V5 (A, B) or against Flag and V5 (C) or V5 only (D). The whole cell lysate (WCL) and the immunoprecipitates (IP) were loaded onto 10% SDS-PAGE gel and Western blotted (WB) using antibodies against c-Myc, V5 or Flag as indicated.

We further tested whether the two morbillivirus V proteins bind to MDA-5, as has been reported for the PIV5 V protein [33–35]. At the same time, we asked whether the C proteins of these morbilliviruses could bind to MDA-5, as there have been suggestions that morbillivirus C proteins play a role in controlling IFN-β induction [18, 41, 49, 61]. We co-expressed MDA-5 with the viral accessory proteins in HEK-293FT cells and observed that only the V proteins bound to MDA-5, as shown by co-precipitation of MDA-5 with immunoprecipitated V and co-precipitation of V proteins with immunoprecipitated MDA-5 (Fig 4A).

### Effect of PPRV V on RIG-I and LGP2

These results indicate that PPRV can block the induction of IFN-β by actively suppressing the activation of the MDA-5-mediated signalling cascade, some or all of which effect is due to the V protein, which binds to MDA-5. Since we also observed an active suppression of the RIG-I-mediated signalling cascade by PPRV (Fig 2), we investigated whether the accessory proteins (V and/or C) were mediating this effect. Co-expression and co-precipitation studies showed that the morbillivirus V proteins, but not the C proteins, bound to RIG-I (Fig 4B); although the interaction appeared to be weaker than the interaction with MDA-5, it was consistently observed across several experiments. There was also a weak interaction between PIV5 V and RIG-I, but this was not consistently observed across experiments.

**Fig 5:**
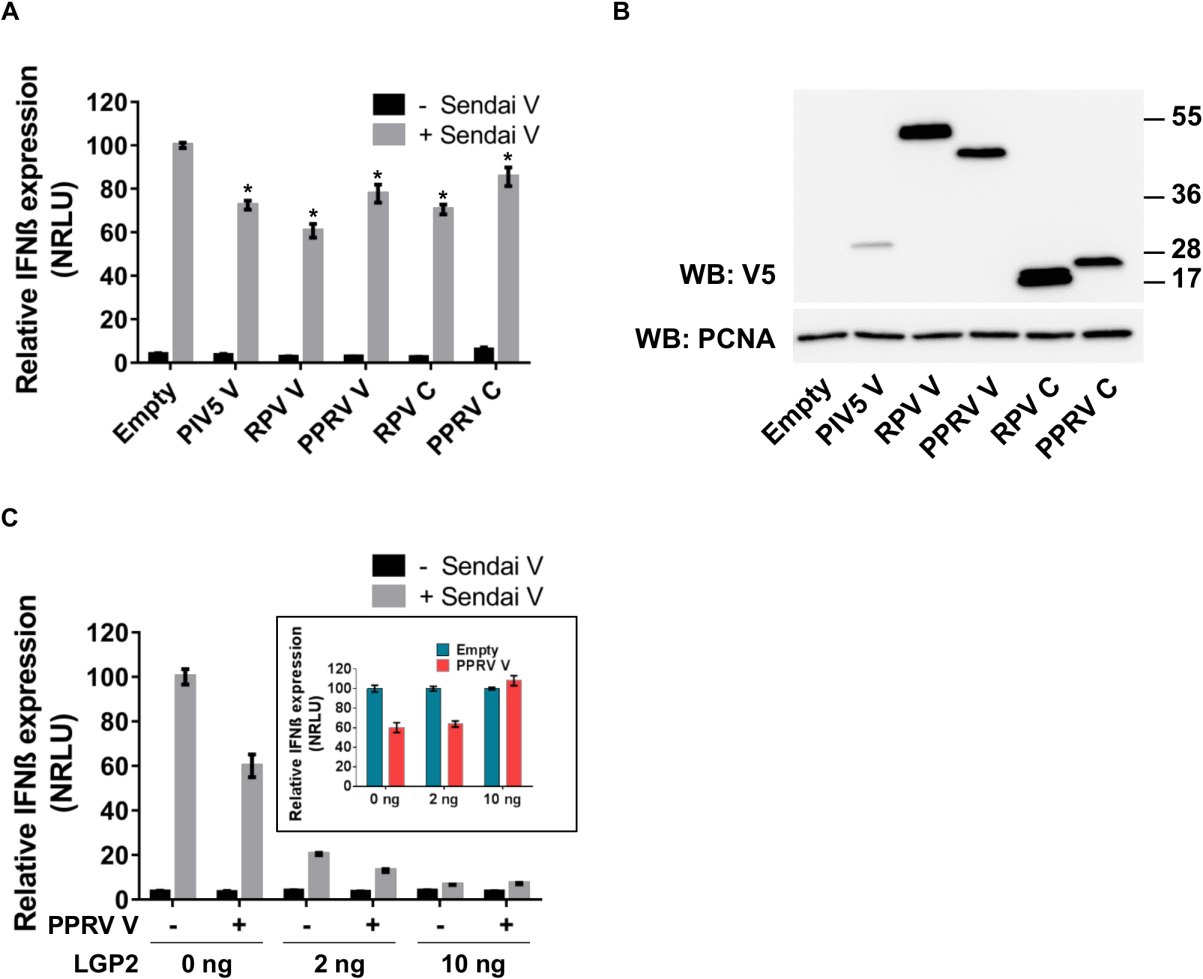
Effect of V and C proteins on the induction of IFN-β by Sendai virus. (A) VHS cells were transfected with pIFN-β-luc (350 ng), pJAT-lacZ (200 ng) and either an empty plasmid (300 ng) or an expression plasmid encoding PIV5 V (300 ng), RPV V (300 ng), RPV C (300 ng), PPRV V (300 ng) or PPRV C (300 ng). At least twenty four hours post transfection, cells were infected with 50 HA units of SeV-DI or left uninfected; 7 hours later the cells were lysed and the cells extracts were assayed for luciferase and β-galactosidase activity. RLU were normalised so that the induction seen in cells transfected with empty vector and infected with SeV-DI was set at 100. Error bars represent the standard error of the mean. ANOVA and Tukey pairwise comparison test were performed to determine under which conditions the SeV-DI-treated cells had lower induction than the control (* = p < 0.05). (B) Cell extracts from parallel transfected wells were run on 10% SDS-PAGE gels and Western blotted with anti-V5 or anti-PCNA (loading control). (C) VHS cells were transfected with the reporter plasmids as above, plus empty plasmid or the expression plasmid encoding PPRV V (500 ng), with increasing amounts of the expression plasmid encoding LGP2 (0 ng, 2 ng and 10 ng). The total amount of DNA was kept constant in all samples by adding empty plasmid as required. Twenty four hours post transfection, cells were infected with 50 HA units of SeV-DI or left uninfected. After 7 hours the cells were lysed and the cell extracts were assayed for luciferase and β-galactosidase activity and RLU values calculated and normalised as described above. (C, Inset) The effect of the expression of the V protein at each level of LGP2 is illustrated by replotting the data shown in (C). The normalized RLU for of each amount of LGP2-transfected/SeV-DI infected cells was used as a reference and set at 100. Error bars represent the standard error of the mean (SEM).

Given the interaction between RIG-I and the V proteins of PPRV and RPV, it was possible that these V proteins could suppress the induction of IFN-β mediated by the activation of RIG-I. We transfected VHS cells with the reporter plasmids and with plasmids encoding the V or the C proteins, and activated the RIG-I signalling cascade by infecting these cells with SeV-DI. All the accessory proteins showed a suppression of the induction of IFN-β, though only to a limited extent (Fig 5A).

The PIV5 V protein has been shown to interact with LGP2. This interaction appears to facilitate the LGP2-mediated inhibition of RIG-I [38]. Co-expression/immunoprecipitation studies showed that PPRV V bound to LGP2 (Fig 4C). However, co-expression of all three proteins (V, LGP2, RIG-I) showed no increase in V-RIG-I co-precipitation in the presence of LGP2, using either human or goat RIG-I (Fig 4D). We also tested whether the presence of extra LGP2 would improve the suppression of the RIG-I-mediated induction of IFN-β in VHS cells expressing PPRV V. The expression of even very small additional amounts of LGP2 had a strong suppressive effect on the RIG-I signalling pathway (Fig 5C), but this over-expression didn’t improve the suppressive effect attributable to the expression of PPRV V (Fig 5C inset).

### Creation of mutant PPRV defective in expression of V or C protein

**Fig 6:**
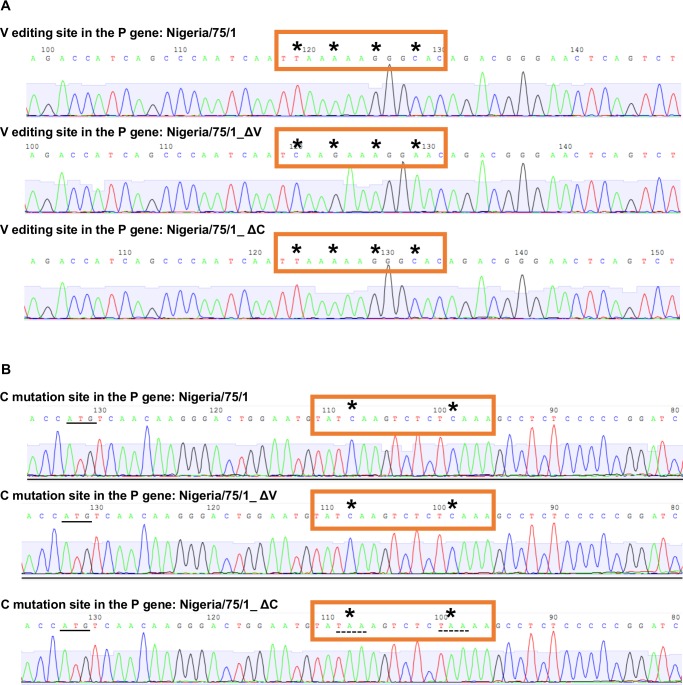
Confirmation of the presence of inserted mutations in the P gene of rescued viruses. (A, B) Viral RNA was extracted from VDS cells infected with PPRV rNigeria/75/1, rNigeria/75/1_ΔC or rNigeria/75/1_ΔV and the P gene of each construct amplified by RT-PCR as described in Methods. (A) Sequence of the P gene from base 723 to 771 showing the V editing site. The specific bases in the editing site mutated to prevent editing are marked (*). (B) Sequence of the P gene from base 79 to 135 showing the start codon for the C protein (underlined) and the mutations introduced into the C ORF to introduce the STOP codons (TAA) (dotted underline) in rNigeria/75/1_ΔC.

These studies suggested a clear role for the PPRV V protein in actively suppressing the production of IFN-β during PPRV infection, suppression achieved through binding to MDA-5 and RIG-I. It was important to confirm this role in the context of viral infection. We therefore made mutant viruses lacking expression of either V (rNigeria/75/1_ΔV) or C (rNigeria/75/1_ΔC), using the reverse genetic system previously described [55]. The morbillivirus V and C proteins are expressed from the P gene through use of, respectively, co-transcriptional editing of the P gene mRNA or translation of an alternate ORF [14, 15]. We made mutations in this gene that were silent in the P protein ORF but prevented expression of one or other of the accessory proteins, as we had previously done for RPV [40]. rNigeria/75/1_ΔV was made by silent changes to the nucleotide sequence in the P gene editing site to prevent the co-transcriptional editing needed to make the V mRNA [16, 17, 40]. rNigeria/75/1_ΔC was made by two base changes that are silent in the P/V ORF but which convert codons 9 and 12 of the C protein ORF to stop codons. Following the rescue of the mutant PPRVs, we confirmed by sequencing that the mutations inserted in the P gene were stable in the rescued virus (Fig 6A and 6B). We characterized the growth of the mutant viruses compared to the parental virus, in both IFN-competent cells (G4 cells) and in IFN-defective cells (VHS cells) to be able to identify any possible effect that the induction of IFN-β may have in these viruses. In addition we looked at the rate of viral protein expression in these two cell types.

**Fig 7:**
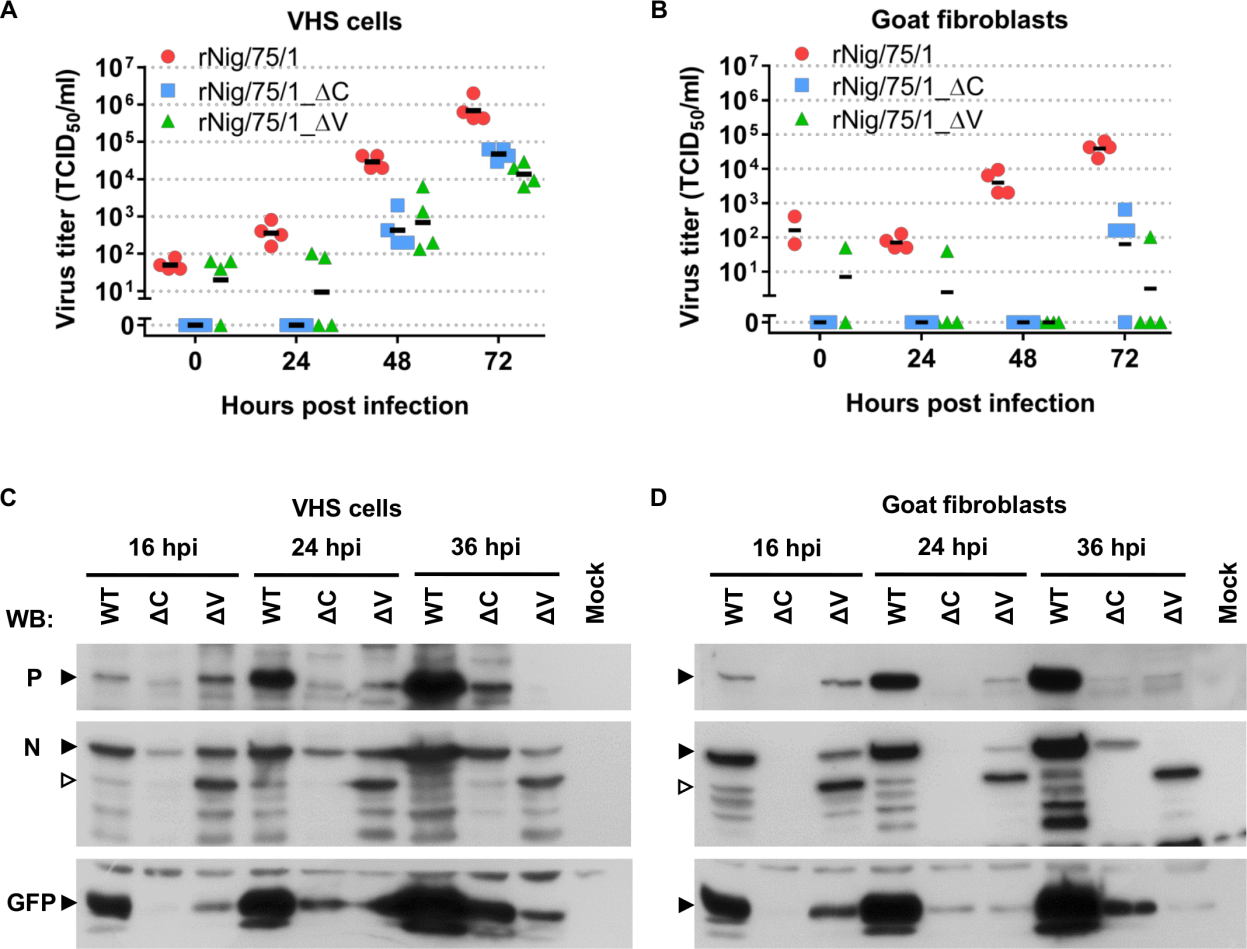
Growth of and viral protein expression by rPPRV in VHS and G4 cells. (A, B) Multi step growth curve of rNigeria/75/1_WT and the mutant viruses rNigeria/75/1_ΔC and rNigeria/75/1_ΔV. VHS cells (A) or G4 (B) cells were infected at a MOI = 0.01. Approximately two hours post infection the virus inoculum was removed and replaced with new medium. At the indicated times, samples of infected cells were frozen along with their medium. The virus titre (TCID_50_) was determined in VDS cells, allowing up to 10 days for signs of cytopathic effect (CPE) or GFP expression. The graph shows the titres for individual samples with the mean of the values marked with a line. The experiment was done twice in duplicate each titrated separately. (C, D) VHS cells (C) or G4 cells (D) were infected with rNigeria/75/1, rNigeria/75/1_ΔC or rNigeria/75/1_ΔV at a MOI = 3 or mock infected. At the indicated times post infection, the cells were lysed and the cell extracts were run on 8% SDS-PAGE gels and Western blotted with a mouse anti-RPV P antibody which cross-reacts with other morbillivirus P proteins, with mouse anti-PPRV N antibody or with rabbit anti-GFP antibody. The positions on the blot of the virally expressed proteins P, N and GFP are indicated by closed arrowheads, and the position of the main proteolytic fragment of N is indicated by the open arrowheads.

Both mutants were able to grow in VHS cells but the replication kinetics of the mutant viruses were slower and reached a final titre at least 1 log lower than the parental virus (Fig 7A). The initial growth of the parental rNigeria/75/1 virus in G4 cells (Fig 7B) was slower than in VHS cells (as seen by the lack of virus growth by 24 hpi), but at subsequent time periods we observed a similar growth rate to that seen for growth in VHS cells. In contrast, the growth of rNigeria/75/1_ΔV was severely impaired in the goat fibroblasts, such that only one of the four attempts to grow the virus in these cells was successful, and the titre achieved was more than 2 logs lower than the titre reached by the parental virus (Fig 7B). The growth of rNigeria/75/1_ΔC was also impaired in the goat fibroblasts; although a measurable titre could consistently be achieved at 72 hpi, it was again more than 2 logs lower than that achieved by the parental virus at this time point.

To see if the defect(s) in the production of the mutant viruses was at the level of viral protein production or at the level of viral assembly, we measured the expression of 3 virally-encoded proteins, N, P and GFP, by Western blot (note that the N protein showed a consistent, time-dependent degradation in the infected cells, leading to the accumulation of both full-length N and fragments). These studies showed that the two mutants had different defects. In both VHS cells and G4 cells infected with rNigeria/75/1_ΔC, there was a much lower expression of all tested proteins at all time points when compared to the expression of these proteins in cells infected with rNigeria/75/1, and this effect was more evident in the G4 cells (Fig 7C and 7D), where the viral proteins N and P were only detected at 36 hpi.

In contrast, in cells infected with rNigeria/75/1_ΔV, the virally-encoded proteins were all clearly detected at 16 hpi, but then failed to increase in amounts with increasing periods of infection. The levels of N and P at 16 hpi were close to those seen in rNigeria/75/1-infected cells at the same time point, but then decreased with time, so that no P, and little or no intact N, could be detected at 36 hpi (Fig 7C and 7D). Levels of GFP in these cells were lower at 16 hpi than for cells infected with the parent virus, and then either stayed approximately the same (VHS cells) or decreased with time in the same way as seen for N and P (G4 cells) (Fig 7C and 7D).

Interestingly, the degradation of N in cells infected with rNigeria/75/1_ΔV showed a different pattern to N protein in cells infected with either the parental virus or rNigeria/75/1_ΔC. The same anti-N-reactive protein fragments are seen for all three viruses, albeit with slightly different sets of fragments in the two cell types. However, the N protein in cells infected with rNigeria/75/1_ΔV predominantly forms a product of 50kDa which is then stable in the cells (open arrowhead, Fig 7). In contrast, in cells infected with the other two viruses, the fraction of N that is degraded was much lower, and the degraded N tended to accumulate as smaller proteins (approx. 32–40 kDa). These observations may be related to the proposed role of the V protein as a chaperone for the N protein during replication [62, 63].

**Fig 8:**
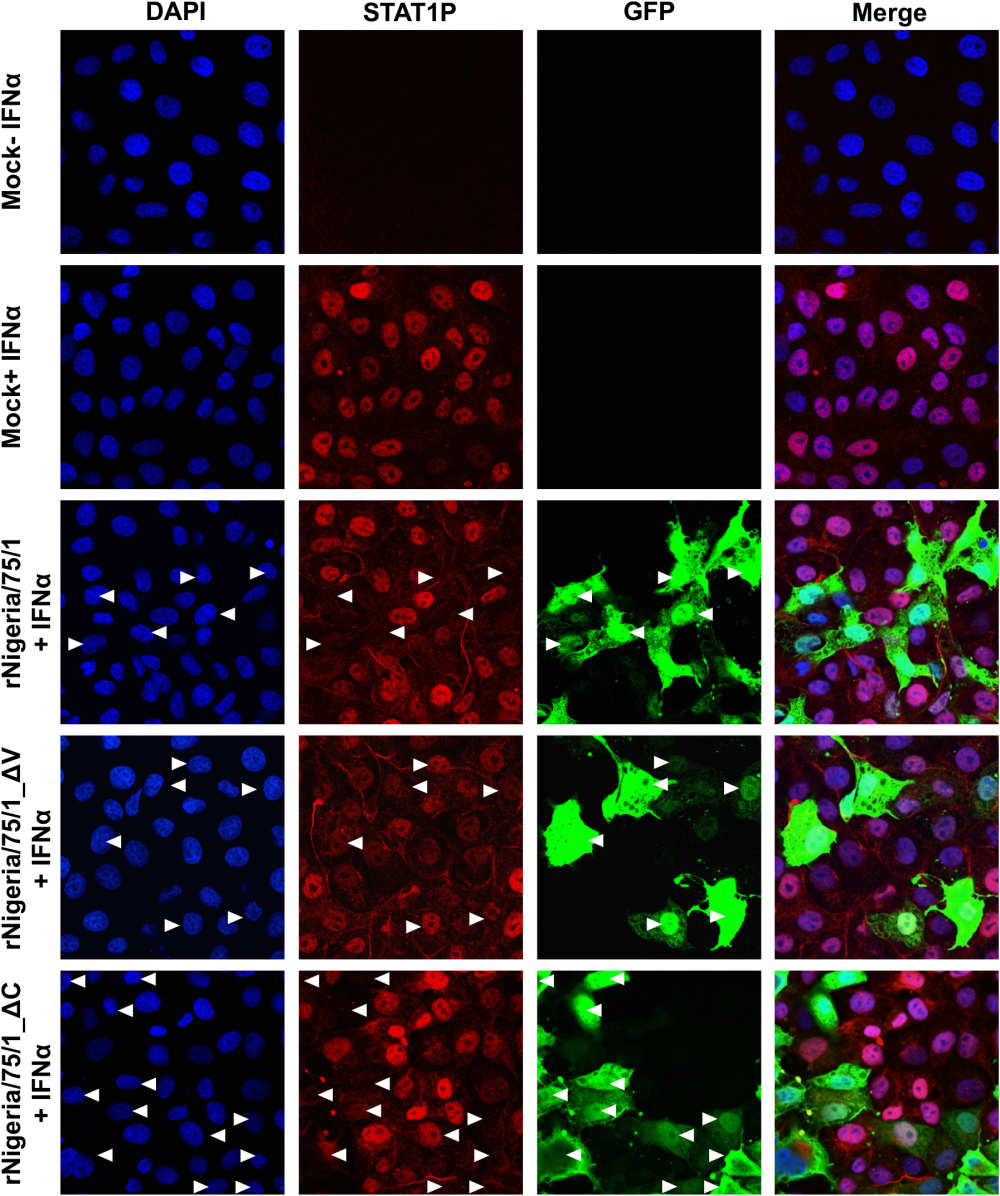
Effect of rPPRV on STAT1 activation. VHS cells were infected with PPRV rNigeria/75/1, rNigeria/75/1_ΔC, rNigeria/75/1_ΔV (MOI ~ 0.5) or left uninfected. At 40 hpi, the cells were treated with 1,000 IU of IFN-α for 30 minutes or left untreated. STAT1P was detected by mouse anti-STAT1P and PPRV was detected using rabbit anti-GFP antibody. Primary antibodies were detected by Alexa Fluor 488 anti-rabbit IgG (green) and Alexa Fluor 568 anti-mouse IgG (red). Nuclei were stained with DAPI. Arrowheads point to the nuclei of infected cells.

Although there are no antibodies available that recognise the PPRV V or C proteins, we confirmed the expression of V protein by rNigeria/75/1_ΔC and its absence in rNigeria/75/1_ΔV by using the known ability of PPRV V protein to block IFN-α-stimulated phosphorylation of STAT1 in infected cells [59]. We infected VHS cells with the parental virus or with one of the two mutants, and left the infection for 18 or 40 hours before treating the cells with IFN-α and labelling intracellular phosphorylated STAT1 (STAT1P) for immunofluorescence (Fig 8). The percentage of infected cells that had STAT1P in the nucleus following IFN-α treatment is presented in Table 1. The phosphorylation of STAT1 was blocked in 60% of cells infected with rNigeria/75/1 by 18 hpi and by 40 hpi almost no infected cells had STAT1P in the nucleus, confirming that infection with the parental virus is blocking the IFN action pathway as previously reported. In cells infected with rNigeria/75/1_ΔC, we observed an approximately 40% block of STAT1 phosphorylation at 18 hpi which increased to 70% by 40 hpi, indicating that the rNigeria/75/1_ΔC was expressing enough V protein to block the phosphorylation of STAT1. On the other hand, most of the cells infected with rNigeria/75/1_ΔV showed IFN-stimulated STAT1P, and this number did not decrease after prolonged infection. These data confirm that this recombinant virus is indeed defective in the expression of the V protein.

**Table 1:** Effect of the mutant PPRVs on the phosphorylation of STAT1.

	Uninfected	rNigeria/75/1_WT	rNigeria/75/1_ΔC	rNigeria/75/1_ΔV
**18 hpi**	99.4	40.0	62.3	85.0
**40 hpi**	100.0	4.0	28.0	85.0

VHS cells were infected with PPRV rNigeria/75/1, rNigeria/75/1_ΔC, rNigeria/75/1_ΔV (MOI ~ 0.5) or left uninfected. At the indicated times, 18 hpi or 40 hpi, the cells were treated with 1,000 IU of IFN-α for 30 minutes or left untreated. The table shows the percentage of infected cells (or uninfected cells in the control group) that showed STAT1 phosphorylation after counting at least 100 cells.

### The role of V and C proteins in the context of PPRV infection

The fact that both the production of progeny virus and the expression of virally-encoded proteins by the mutant viruses was more defective in the IFN-producing goat fibroblasts than in the VHS cells is consistent with both accessory proteins of PPRV playing a role in the evasion or suppression of type I IFN responses. To further investigate this possibility, we measured the induction of IFN-β over the first 24 h after infection of VHS and G4 cells with the mutant PPRVs (MOI = 1); this was done by measuring IFN-β promoter activation using reporter assays and levels of IFN-β mRNA using RT-qPCR, as described for previous experiments.

**Fig 9:**
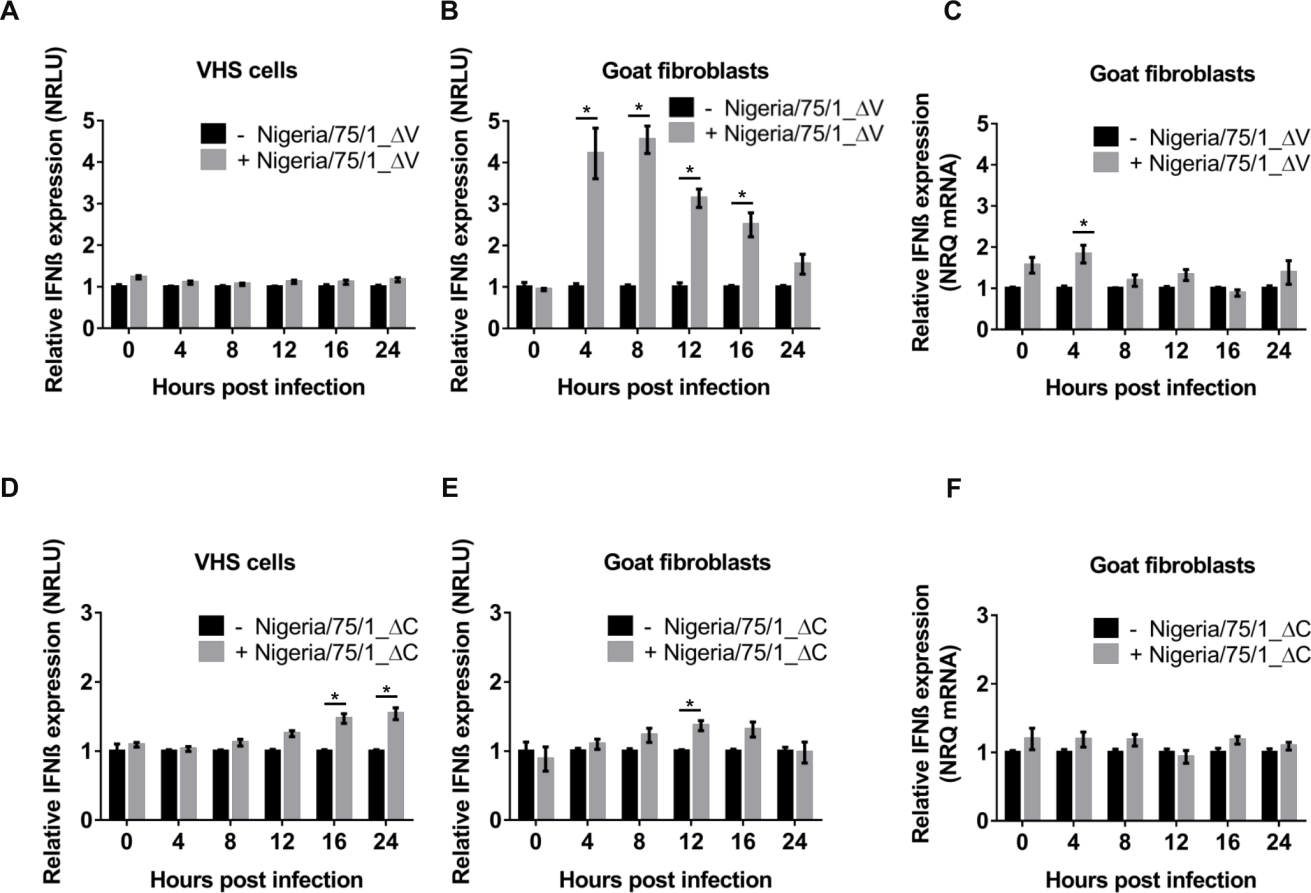
IFN-β induction during the infection of VHS cells or G4 cells with PPRV lacking V or C expression. VHS cells (A, D) or G4 cells (B, E) were transfected with reporter plasmids as described in for Fig 1. At least 18 hours post transfection, cells were infected with rNigeria/75/1_ΔV, rNigeria/75/1_ΔC (MOI = 1) or left uninfected. At each indicated time after infection, samples of cells were lysed and the cell extracts were assayed for luciferase and β-galactosidase activity. RLUs were normalised over time and between experiments by setting the value for uninfected cells to one. (C, F) G4 cells were infected with rNigeria/75/1_ΔV, rNigeria/75/1_ΔC (MOI = 1) or left uninfected and the cells lysed at the specified time points to extract total RNA. RT-qPCR was performed as described in Methods and the normalized relative quantities (NRQ) of the IFN-β mRNA calculated relative to the geometric mean of the amount of SDHA and GAPDH mRNA. The graphs show the relative NRQ of infected cells compared to uninfected cells, set to 1 at each time point. The error bars represent the standard error of the mean (SEM). The ANOVA test and Tukey pairwise comparison test were used to determine the significance of differences between the means (* = p < 0.05).

Since the PPRV V protein, as for other paramyxovirus V proteins, blocks IFN-β induction, we predicted that the ΔV virus would show a significant difference to the virus expressing the V protein. However, this difference was minor and was only apparent in the cells that could produce interferon. VHS infected with rNigeria/75/1_ΔV, like those infected with the parental rNigeria/75/1 virus, showed no activation of the IFN-β promoter on the reporter plasmid (Fig 9A; cf Fig 1D). In G4 cells transfected with the reporter plasmids, the transient activation of the IFN-β promoter observed previously in cells infected with rNigeria/75/1 (Fig 1E) was again observed, but was quantitatively greater (Fig 9B), and so could be detected earlier (4 hpi) and was still significant at 16 hpi, though decreasing. Reflecting this stronger induction of IFN-β, we could detect an increase in the amount of IFN-β mRNA in rNigeria/75/1_ΔV-infected G4 cells (at 4 hpi) (Fig 9C), whereas this was not seen in the cells infected with the parent virus (Fig 1F).

Interestingly, although this virus still produces V protein, infection with rNigeria/75/1_ΔC showed weak but clear induction of IFN-β during the first 24 hpi. This was visible in both VHS or G4 cells transfected with the reporter plasmids, albeit with slightly different kinetics, being detectable at 12 hpi in the fibroblasts, but not until 16 hpi and 24 hpi in the VHS cells (Fig 9D and 9E). As expected from this weak induction of the reporter gene, no significant change in the levels of IFN-β mRNA was detected (Fig 9F).

**Fig 10:**
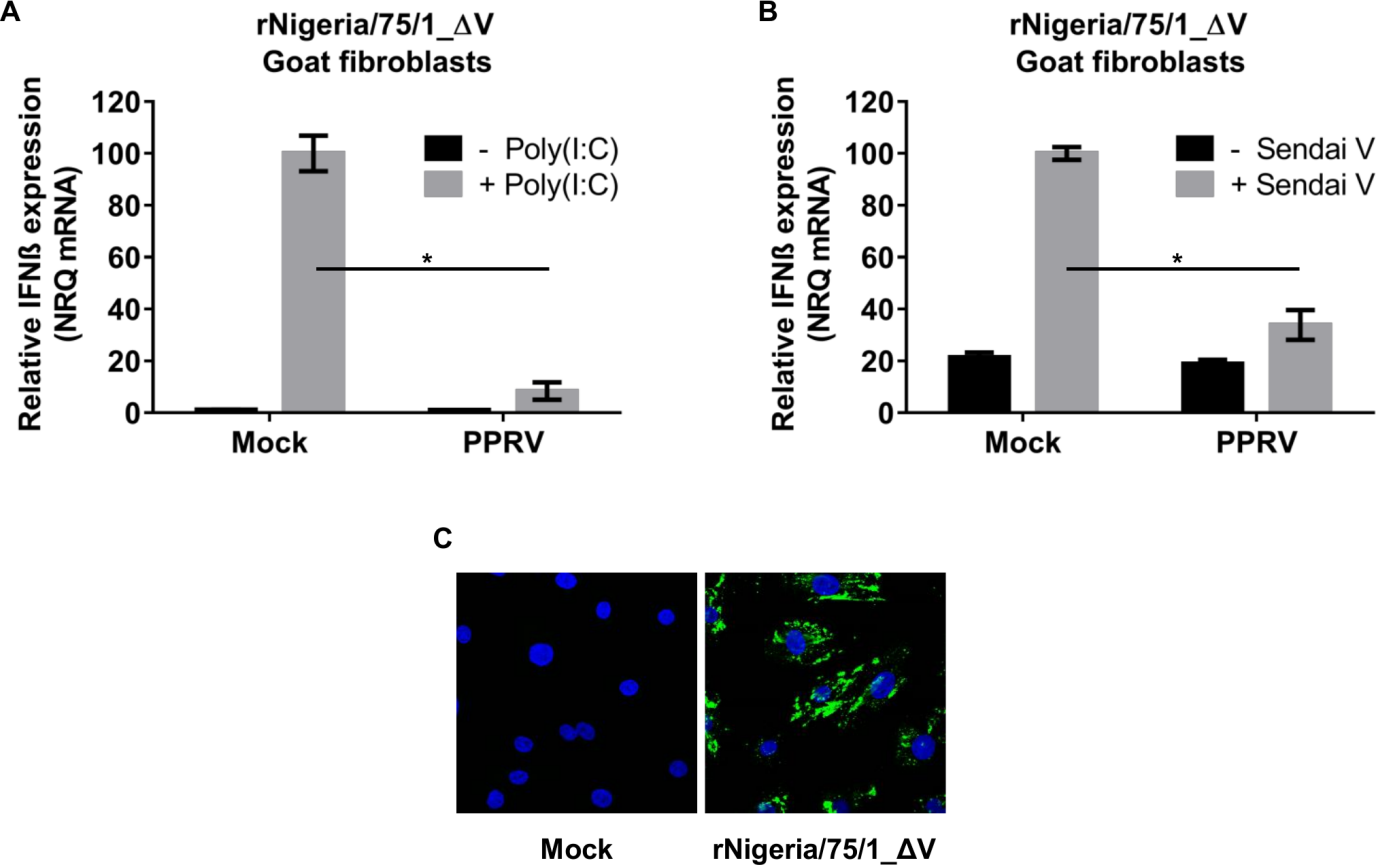
The V protein of PPRV is not essential in the context of virus infection to block the induction of IFN-β by poly(I:C) or Sendai virus. (A, B) G4 cells were left uninfected or infected with rNigeria/75/1_ΔV (MOI = 3) for 16 hours before (A) transfection with 1 μg of poly(I:C) or (B) infection with 50 HA units of SeV-DI. At 5 hours after poly(I:C) transfection or 7 hours after SeV-DI infection the cells were lysed to extract total RNA. RT-qPCR was performed as described in Methods and the normalized relative quantities (NRQ) of the IFN-β mRNA calculated relative to the geometric means of the SDHA and the GAPDH mRNA. The NRQ was normalised between experiments by setting treated-uninfected cells to 100. The error bars represent the standard error of the mean (SEM). The ANOVA test and Tukey pairwise comparison test were used to determine the significance of differences between the means (* p = < 0.05). (C) G4 cells from parallel infected cells on coverslips were fixed in 3% PFA, permeabilized using 0.2% Triton and blocked with 0.2% gelatine in PBS. PPRV was detected using mouse anti N antibody followed by Alexa Fluor 488anti-mouse (green). Nuclei were stained by DAPI. Images were collected using the 63x magnification lens.

We then compared the abilities of the mutant viruses to inhibit IFN-β induction through either the MDA-5 or the RIG-I-mediated signalling cascade. The results shown in Fig 9 suggest that rNigeria/75/1_ΔV is still able to affect the induction of IFN-β, albeit less effectively than the parental virus, as the initial induction in G4 cells is suppressed over time, similar to the pattern seen in cells infected with rNigeria/75/1. In G4 cells infected with rNigeria/75/1_ΔV (MOI = 3), the induction of IFN-β by transfection with poly(I:C) (Fig 10A) or infection by SeV-DI (Fig 10B) was indeed blocked, indicating that the V protein of PPRV is not the sole viral protein actively blocking the activation of the IFN-β promoter.

**Fig 11:**
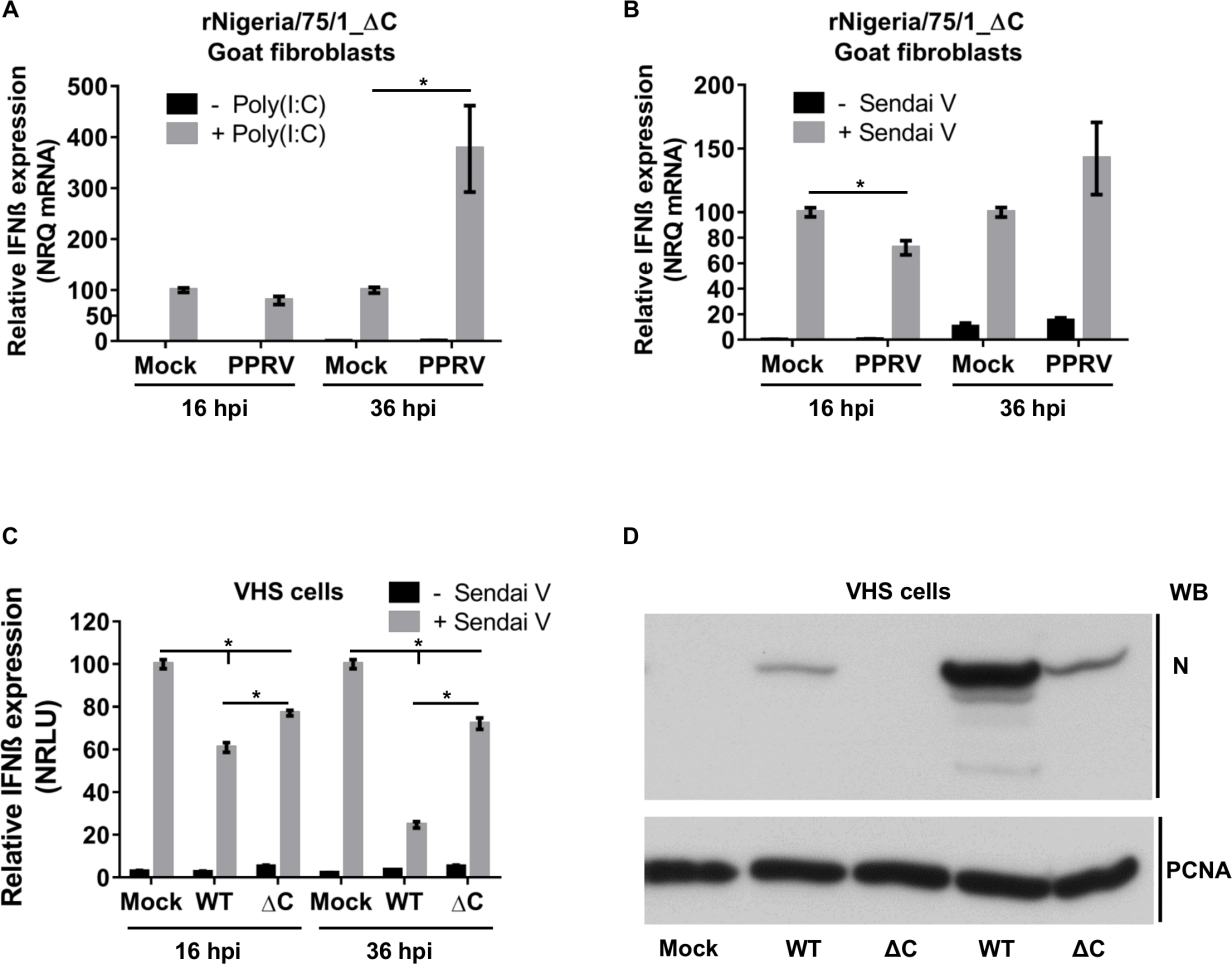
The C protein is necessary during PPRV infection for maximal inhibition of induction of IFN-β by either poly(I:C) and Sendai virus. (A, B) G4 cells were left uninfected or infected with rNigeria/75/1_ΔC (MOI = 3) for 16 or 36 hours before (A) transfection with 1 μg of poly(I:C) or (B) infection with 50 HA units of SeV-DI. At 5 hours after poly(I:C) transfection or 7 hours after SeV-DI infection cells were lysed to extract total RNA. RT-qPCR was performed as described in Methods and the normalized relative quantities (NRQ) of the IFN-β mRNA calculated relative to the geometric means of the SDHA and the GAPDH mRNA. The NRQ was normalised between experiments by setting treated-uninfected cells to 100. (C) VHS cells were transfected with the reporter plasmids as described for Fig 1. At least 18 hours post transfection, cells were infected with rNigeria/75/1 (WT), with rNigeria/75/1_ΔC (ΔC) (MOI = 3) or left uninfected. At 16 or 36 hpi cells were infected with 50 HA units of SeV-DI or left uninfected. At 7 hours after SeV-DI infection the cells were lysed and the cell extracts were assayed for luciferase and β-galactosidase activity. RLUs were normalised between experiments by setting treated-uninfected cells to 100. (A-C) The error bars represent the standard error of the mean (SEM). The ANOVA test and Tukey pairwise comparison test were used to analyse differences between the means (* = p < 0.05). (D) VHS cell extracts from parallel infected/uninfected cells treated as in (C) were run on 10% SDS-PAGE gels and Western blotted with mouse anti-PPRV N antibody or with mouse anti-PCNA (loading control).

The time course studies with rNigeria/75/1_ΔC showed lower and/or later induction of IFN-β in cells infected with this virus. This may be due to the slower growth kinetics of this mutant virus, which delay the induction of IFN-β. When we infected the goat fibroblasts with rNigeria/75/1_ΔC and treated them with either poly(I:C) or SeV-DI at 16 hpi, inhibition of induction of IFN-β mRNA was only significant in SeV-DI infected cells (Fig 11A and 11B). In order to compensate for the impaired growth and protein expression of rNigeria/75/1_ΔC, we allowed the infection to proceed for 36 hours before treating the cells to induce IFN-β. However, in these circumstances, we found that there was an increase in the induction of IFN-β mRNA in infected cells rather than any inhibition. This may be due to a positive feedback effect, whereby IFN-β induced during the first 36 hours of infection (Fig 9) causes the cells to be super sensitive to the stimulating agent. To eliminate any effect of endogenously produced type I IFN, we repeated the study in VHS cells, transfected with reporter plasmids as previously. With this system we could show that the rNigeria/75/1_ΔC virus was able to significantly inhibit the induction of transcription from the IFN-β promoter at 16 hpi, though its effect was not as strong as for the parental virus (Fig 11C). At 36 hpi, both viruses still blocked activation of the IFN-β promoter, and no sensitization was observed, but only the parental virus showed an increased blockade at the later time, while that resulting from infection with rNigeria/75/1_ΔC was essentially unchanged (Fig 11C), despite the higher level of viral protein in the cells at 36 hpi compared to 16 hpi (Fig 11D).

## Discussion

Paramyxoviruses, as with many viruses, have evolved to be poor inducers of IFN-β. Initial induction of IFN-β is thought to be through activation of RIG-I by the small leader RNA produced during mRNA transcription [64, 65]. However, because of their mode of replication, progeny genomes are encapsidated, preventing the formation of dsRNA [66] until later in infection when DIs [67] and dsRNA [68] are produced in significant amounts. In addition to such avoidance measures, the viruses have mechanisms to actively interfere with the induction of IFN-β. These mechanisms have been associated chiefly with the viral V protein through its interaction with MDA-5 [33–35] and possibly LGP2 [37, 38]. In addition, MeV V has been reported to interact with PP1 [36] and act as a decoy substrate to inhibit interferon induction through Toll-like receptors [69], while several paramyxovirus V proteins bind to IRF3 [70]. Although there is strong evidence for these additional functions of the paramyxovirus V protein from assays with individually expressed proteins, there have been relatively few studies of the significance of these interactions during viral infection.

We have created recombinant PPRVs that are defective in the expression of either V or C protein. Both mutant viruses presented differences in growth and protein expression compared to the parental virus. These defects were much more severe than those seen with similar recombinant RPV or MeV [40, 71, 72], where ΔV or ΔC vaccine strain viruses replicated reasonably well in cell culture, although ΔC viruses have shown some level of defect in replication in most cases [40, 43, 73, 74]. Notably, the double knockout mutant of RPV was viable, whereas the individual defects in PPRV_ΔV and ΔC were such that we were unable to recover the double mutant. Both ΔV and ΔC mutations, in RPV and MeV, led to differences in viral RNA synthesis and protein translation [40, 43, 73, 75] as well as in replication *in vivo* [76, 77]. Similar to our findings with PPRV_ΔC, RPV and MeV lacking C expression have shown reduced viral protein expression [40, 43]; in contrast to RPV_ΔV or MV_ΔV, which showed increased viral RNA or protein expression [40, 75], PPRV_ΔV was defective in both growth and viral protein expression, even in Vero cells, which lack intrinsic expression of type I IFNs. We noted that viral protein expression in cells infected with PPRV_ΔV was initially at near wild type levels, but appeared to stop at around 18 hpi, since the amount of viral protein detected decreased, as might be expected due to natural turnover of the proteins, while the levels in cells infected with PPRV or PPRV_ΔC continued to increase. It is not possible to determine the cause of this underlying difference from the available data, and it will be important in the future to identify the cause of this growth defect in PPRV_ΔV.

We confirmed the finding predicted by previous studies on paramyxoviruses [33, 34] that the PPRV V protein binds to MDA-5 and actively blocks the induction of IFN-β mediated through this RLR protein. Unexpectedly, the induction of IFN-β in cells infected with the V-deficient virus was effectively controlled, in a similar manner to that observed in cells infected with the parental virus. This indicates that PPRV has a V-independent mechanism for actively inhibiting IFN-β induction. The observation that both RIG-I-mediated and MDA-5-mediated pathways are blocked in PPRV_ΔV-infected cells suggests that this mechanism acts at or after the level of mitochondrial antiviral-signalling protein (MAVS). The C protein of MeV has been shown to block IFN-β induction [49], as has that of SeV [78], and in the former case this activity took place in the nucleus, but in neither case was the mechanism explored in infected cells. The expression of other inhibitors of IFN-β induction by PIV5 has been suggested [67], although these authors stated that their mutant virus, which expressed a defective V, did not block the induction of IFN-β by other agents.

An interesting observation was that, while neither PPRV nor the ΔV mutant activated the IFN-β promoter in Vero cells, both did so in the goat fibroblasts, and at a relatively early stage in the infection cycle. There is evidence [64] that the naked leader RNA produced during paramyxoviral mRNA transcription can induce IFN-β, and our observations are in accord with such an early stimulation which is then suppressed by the synthesis of V protein and/or another viral protein, possibly C. The absence of induction in the Vero cells probably reflects their reduced baseline amounts of IFN-induced proteins, since these cells have no constitutive autocrine stimulation to maintain levels of these proteins [79, 80]. Compared to the parental virus, the V-defective virus induced slightly higher levels of IFN-β during this early stage. Given the slow growth of PPRV_ΔV, even in Vero cells, this observation may be explained by an increased level of DI particles in the stocks of the mutant virus, since DIs appear to accumulate during prolonged culture of paramyxoviruses [67].

Several studies have suggested that the C protein of morbilliviruses is involved in viral RNA transcription and its absence leads to alterations in the amount and nature of viral RNA synthesis [40, 42, 81–83]. It has also been shown that viruses defective in C protein show increased amounts of dsRNA, which can act as an inducer of IFN-β [43, 47]. We were not able to show any difference in the amount of dsRNA in PPRV-infected and PPRV_ΔC-infected cells by immunofluorescence (S3 Fig), but this may be due to limitations of the sensitivity of the assay used. We found a late induction of IFN-β in cells infected with PPRV_ΔC, and an apparent super-sensitisation of IFN-producing cells to IFN-inducing agents, observations which are in accord with a hypothesis that the absence of C protein in this virus also leads to the production of small amounts of dsRNA, which activate the IFN-β promoter, possibly through PKR [44]. In cells which do not produce IFN, we found that the ΔC virus was able to inhibit IFN induction, presumably through the synthesis of V protein. Interestingly, the strength of this inhibition did not increase from 16 hpi to 36 hpi. It is possible that the V protein is less stable than the viral proteins assayed [84]. Alternatively, the accumulation of dsRNA in PPRV_ΔC-infected cells may be having sensitisation effects even in the Vero cells.

An interesting observation was the weak but consistent interaction between the PPRV and RPV V proteins and RIG-I. While a direct interaction of a morbillivirus V protein with RIG-I was not reported before [34], an indirect interaction has been shown for the V proteins of PIV5 and SeV [38], an interaction mediated by that between the V proteins and LGP2, which in turn binds to RIG-I. We also demonstrated here an interaction between PPRV V and LGP2, but in our case co-expression of LGP2 didn’t increase the interaction between PPRV V and RIG-I, nor did it improve the inhibition of RIG-I-mediated IFN-β induction by PPRV V protein. It is most likely that, in this case, the interaction is direct, but of lower affinity than that between PPRV V and MDA-5. Further work will be required to determine if this low affinity interaction between PPRV and RIG-I is related to the effect of the V protein on RIG-I-mediated induction of IFN-β.

Paramyxovirus V proteins bind to MDA-5 and LGP2 through a domain on these RLRs known as the minimal V binding region (MVBR), initially characterized as being between amino acids 327 and 465 of LGP2 in one study, or 351–479 in another [37, 38]. While the work presented here was on-going, detailed dissection of the MVBR [85, 86] has identified specific amino acids which are important for this interaction, notably MDA-5 R806/LGP2 R455, which is a leucine at the equivalent position in RIG-I. Interestingly, mutating LGP2 R55 to a leucine abolished most of the binding to MeV V but not that to Nipah virus V or PIV5 V [86]. Examining our fibroblast-derived LGP2 clone in the light of these data showed that it had a cysteine instead of an arginine at position 455; however, this substitution had clearly had no effect on the binding of this LGP2 to PPRV V, nor on the ability of the LGP2 to inhibit RIG-I-mediated IFN-β induction. This provides further support for the proposal that the detailed mechanisms of the interactions between V proteins and these helicases vary with different viruses [86].

The results presented in this study support continued investigation of the morbillivirus C proteins and their effects on signal transduction, and on the effects of the interaction between the RLRs and the V proteins other than the induction of IFN-β. In addition, while these data reflect what may be the effects of PPRV V and C proteins when the virus infects non-immune cells, more studies are needed to understand how these viruses may block the induction of IFN-β in their primary targets *in vivo*, the immune cells bearing the virus receptor, SLAM.

## Materials and methods

### Cells and viruses

Vero cells expressing the human form of the morbillivirus receptor (SLAM) (VHS), Vero cells expressing canine SLAM (VDS) and HEK-293FT cells were maintained as previously described [59]. G4 goat skin fibroblasts were the gift of Dr T Barrett (The Pirbright Laboratory) (now deceased), and were maintained in Iscove’s Modified Eagle’s medium (IMDM) containing 10% FCS, penicillin (100 U/ml) and streptomycin (100 μg/ml).

The field strains of PPRV used in these studies, Nigeria/76/1, Ivory Coast/89 and Sudan/Sinnar/72, have been previously reported [9], and were used at the first (Ivory Coast/89) or second (Nigeria/76/1, Sudan/Sinnare/72) passage in VDS cells. The recombinant version of the vaccine strain of PPRV (rNigeria/75/1) has been previously described [55]. The mutant viruses rNigeria/75/1_ΔV and rNigeria/75/1_ΔC were made using the same full-length genome construct in which the PPRV P gene was engineered to prevent the expression of either the C or the V protein. Preventing C protein expression was done by changing 2 codons in the first 12 of the C protein ORF to stop codons, without changing the P/V protein ORF; preventing V protein expression was done by a set of 4 silent changes to the gene sequence to abolish the co-transcriptional editing site [40]. In each case, a two-step overlapping PCR approach was used to simultaneously amplify and mutate a section of the PPRV genome including the whole of the P gene and bracketed by unique *Acc*III and *Not*I sites. When the sequence of the mutated construct was confirmed, the *Acc*III-*Not*I fragment was cut out and used to replace the equivalent section from the full genome plasmid. The new viruses were recovered as previously described [55] using these mutated genome plasmids. All recombinant viruses were used at the second passage in VDS cells. The sequences of the primers used are available from the authors on request.

All virus titres were determined as the 50% tissue culture infectious dose (TCID_50_). Titrations were carried out on VDS cells and calculated using the Spearman-Kärber method [87].

### Plasmids

The expression plasmids pJAT-lacZ [88], pIFΔ(-116)lucter [89] (pIFN-β-luc), pEF-PIV5-V [90], pEF-RIG-I_c-Myc, pEF-MDA-5_c-Myc, pEF-ΔCARD_RIG-I and pEF-ΔCARD_MDA-5 [35] have been described previously. The expression plasmids for the V and C proteins of RPV/Saudi/81 (pcDNA-RPV-V-V5 [58] and pcDNA-RPV-C-V5 [91]) and the V protein of the wild type PPRV/Turkey/2000 isolate (pcDNA-PPRV-V-V5 [59]) have also been described. The corresponding expression construct for the PPRV/Turkey/2000 C protein (pcDNA-PPRV-C-V5) was made by amplifying the C protein ORF from a clone of the P gene and inserting it into pcDNA6/V5/His. The expression plasmids pCAGGS-Flag-goat-LGP2 and pCAGGS-cMyc-goat-RIG-I were made by amplifying the LGP2 and RIG-I ORFs from cDNA prepared from total RNA extracted from G4 cells, and cloned into a mammalian expression plasmid (pCAGGS) in frame with either a 5’ Flag or 5’ c-Myc epitope tag. Primers for the amplification of the goat proteins were designed based on consensus sequences for goat LGP2 and RIG-I created by running a BLAST search with the equivalent sheep and cattle nucleotide sequences on available high throughput genomic sequences (HTGS) from goats (*Capra* spp.). PCRs were performed using a proofreading polymerase (KOD; Novagen) and the PCR products introduced into plasmids were sequenced completely.

### Antibodies and other reagents

The strongly interferon-inducing preparation of the Cantell strain of SeV (SeV-DI) [92] was purchased from Charles River Laboratories; the virus was diluted in cell medium, added to the cells and after two hours the virus suspension was removed and new medium was added to the cells for the remaining of the incubation period. Infection of cells with SeV-DI was confirmed by immunofluorescence using an antibody against SeV NP protein (1:100) (mouse monoclonal, clone 877) kindly provided by Prof Claes Örvell (Karolinska University Laboratory, Sweden).

Poly(I:C) (Amersham Biosciences) was transfected into cells using TransIT-LT1 (Mirus Bio LLC) for VHS cells or Lipofectamine 2000 (Invitrogen) for G4 cells. All plasmid transfections were carried out using TransIT-LT1 according to the manufacturer’s recommendations.

Mouse anti-RPV P (2–1) [93] was the kind gift of Dr Sugiyama. Other antibodies used for immunofluorescence, Western blot and immunoprecipitation were: mouse anti-V5 and horseradish peroxidase (HRP)-conjugated mouse anti-V5 (AbD Serotec), mouse anti-c-Myc tag (clone 4A6) (Upstate®, Merck Millipore), mouse anti-c-Myc tag (clone 9E10) (Roche Diagnostics), mouse anti-Flag tag (clone M2) (Sigma-Aldrich,), mouse anti- proliferating cell nuclear antigen (PCNA) (PC10) (Santa Cruz Biotechnology), HRP-conjugated sheep anti-mouse IgG and HRP-conjugated donkey anti-rabbit IgG (GE Healthcare), mouse anti-PPRV N (CIRAD-EMVT), rabbit anti-GFP (Abcam), mouse monoclonal antibody against phosphotyrosine 701-STAT1 (BD Biosciences), Alexa Fluor® 488-goat anti-rabbit IgG and Alexa Fluor® 568-goat anti-mouse IgG (Thermo-Fisher).

### Co-immunoprecipitation, SDS-PAGE and western blots

HEK-293FT cells seeded in 6 well-plates were transfected with various expression plasmids as described in the figure legends and incubated for 48 hours. Cell lysis, immunoextraction, SDS-PAGE and Western blot analysis were as previously described [94].

### Immunofluorescence assay of STAT1 phosphorylation

Interferon-induced phosphorylation of STAT1 in infected cells was determined essentially as previously described [59], except that VHS cells were used, and infected cells were detected using rabbit anti-GFP, since all the constructs express GFP from an extra transcription unit [55].

### Luciferase reporter assays

Cells in 12-well plates were transfected with the reporter plasmid pIFN-β-luc along with the transfection control plasmid pJAT-lacZ; other plasmids transfected at the same time are described in the figure legends. Cells were incubated for different periods after transfection depending on the experiment. Luciferase and β-galactosidase assays were carried out as previously described [59]. Luciferase readings were normalized to β-galactosidase readings for each well as relative light units (RLUs). Activation of the IFN-β promoter was calculated relative to the relevant control, indicated in the figure legends.

### RNA extraction, reverse transcription and real-time qPCR for IFN-β mRNA analysis

RNA was extracted from G4 cells seeded in 24-well plates, using the RNeasy Mini Kit (Qiagen), diluted in RNase-free water and digested with 2 U of recombinant DNase I (TURBO™ DNase, Ambion) for 2 hours at 37°C. Reverse transcription (RT) was made from 200 ng of total RNA using SuperScript® II RT (Invitrogen™), primed with oligo(dT)-Anch ((T)_16_VN). Parallel aliquots of RNA were processed in the same way without adding the reverse transcriptase (RT-) as a control to detect the amplification of genomic DNA in the samples. Samples from the RT reaction were diluted 1:3 with DNase/RNase-free water (Gibco) and quantitative real-time PCR (RT-qPCR) was performed using SYBR®Green PCR Master Mix (Life Technologies) in a Rotor-Gene 3000 (Corbett Life Science) or Stratagene Mx3005P (Agilent). RT- and no-template controls (NTC) were also run in the qPCR. The PCR cycling conditions were 95°C for 10 minutes and 40 cycles of 95°C for 15 seconds, 60°C for 30 seconds and 72°C for 30 seconds, followed by a melt curve. The mean threshold cycle number (Ct value) for two housekeeping genes (glyceraldehyde phosphate dehydrogenase (GAPDH) and succinate dehydrogenase A (SDHA)) were used to normalize samples, and the efficiency of the reaction was calculated by a calibration curve with serial dilutions of cDNA. Individual mRNAs were assayed in duplicate, and single experiments performed in triplicates. The normal relative quantities (NRQ) of IFN-β were calculated by the formulas described in [95] and [96].

## Ethics statement

The primary goat fibroblast cells were prepared from samples collected during animal studies that were carried out under licences issued by the UK Home Office in accordance with relevant legislation, and after approval by the Institute for Animal Health Ethical Review Committee (precursor to the current Pirbright Institute Animal Welfare and Ethical Review Board).

## Supporting information

S1 Fig Pathway determination for Sendai virus and poly(I:C)-mediated induction of IFNβ. (TIFF)

S2 Fig Superinfection of PPRV-infected cells with Sendai virus. (TIFF)

S3 Fig dsRNA detection in cells infected with PPRV or mutant PPRV. (TIFF)
