# Supplementary figures and images for: Control of the induction of type I interferon by Peste des petits ruminants virus
Source: PLoS One. 2017 May 5;12(5):e0177300. doi: 10.1371/journal.pone.0177300 (PMC5419582; doi:10.1371/journal.pone.0177300)

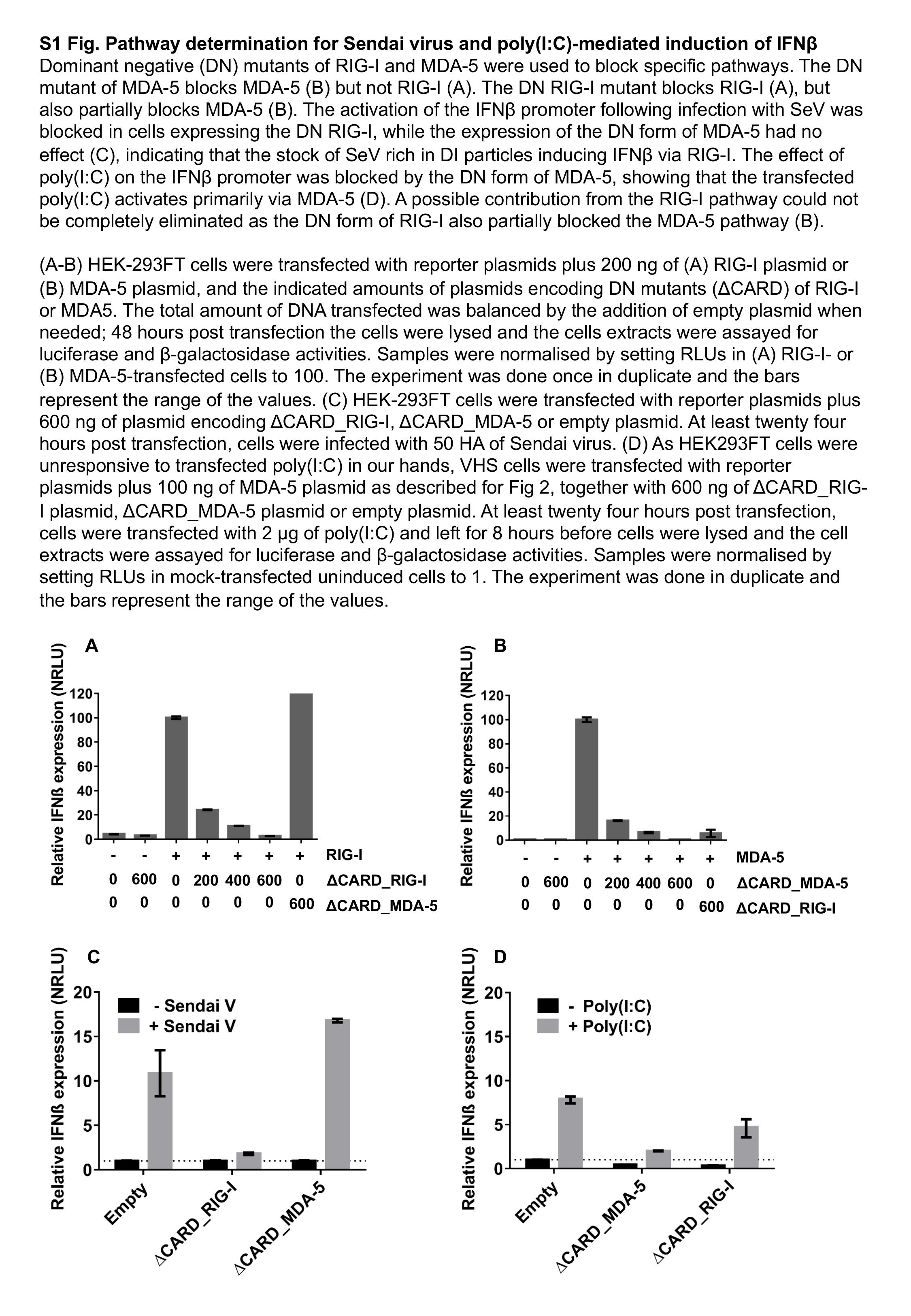

Supplement: S1 Fig — (TIFF) [file pone.0177300.s001.tiff]

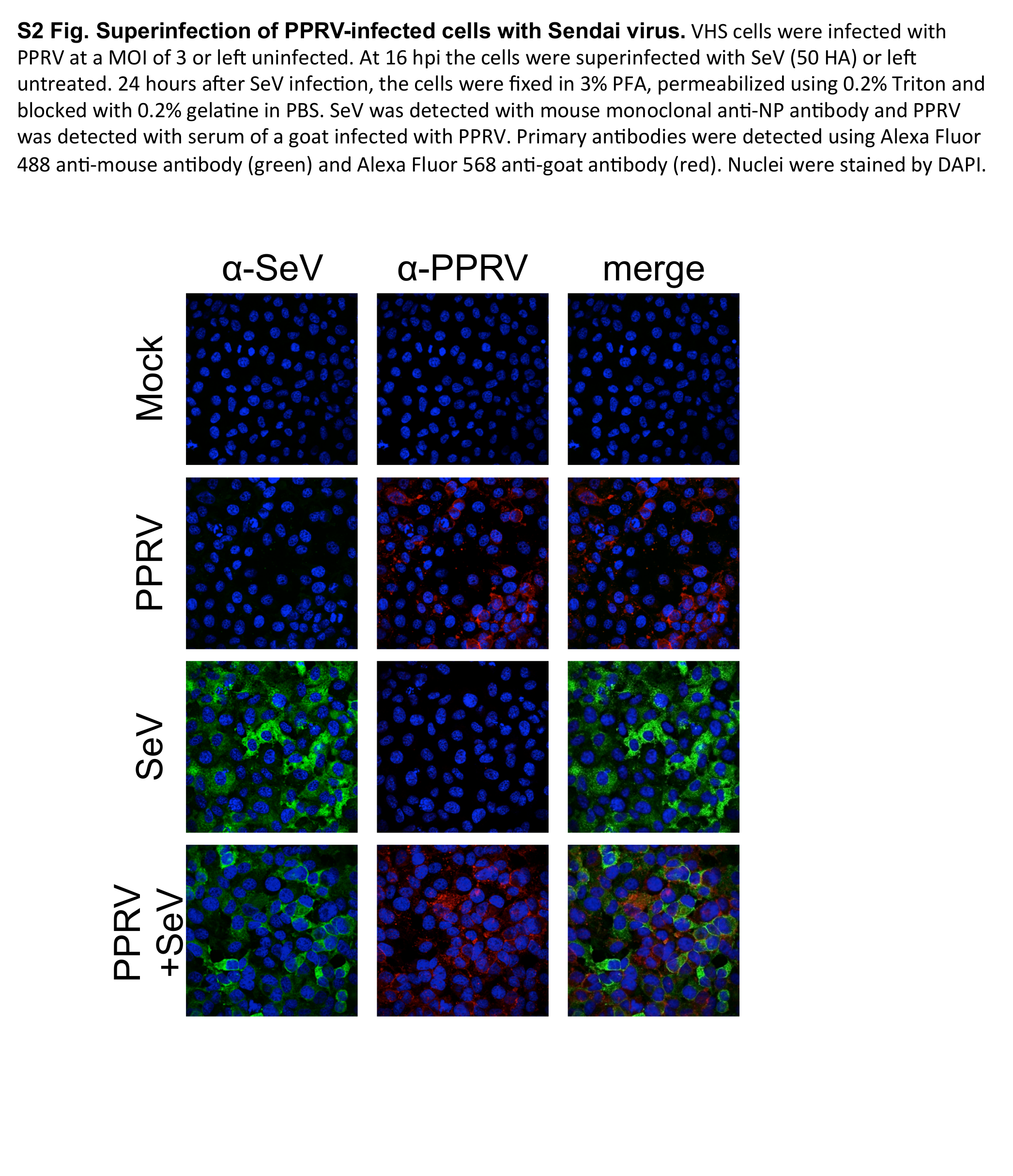

Supplement: S2 Fig — (TIFF) [file pone.0177300.s002.tiff]

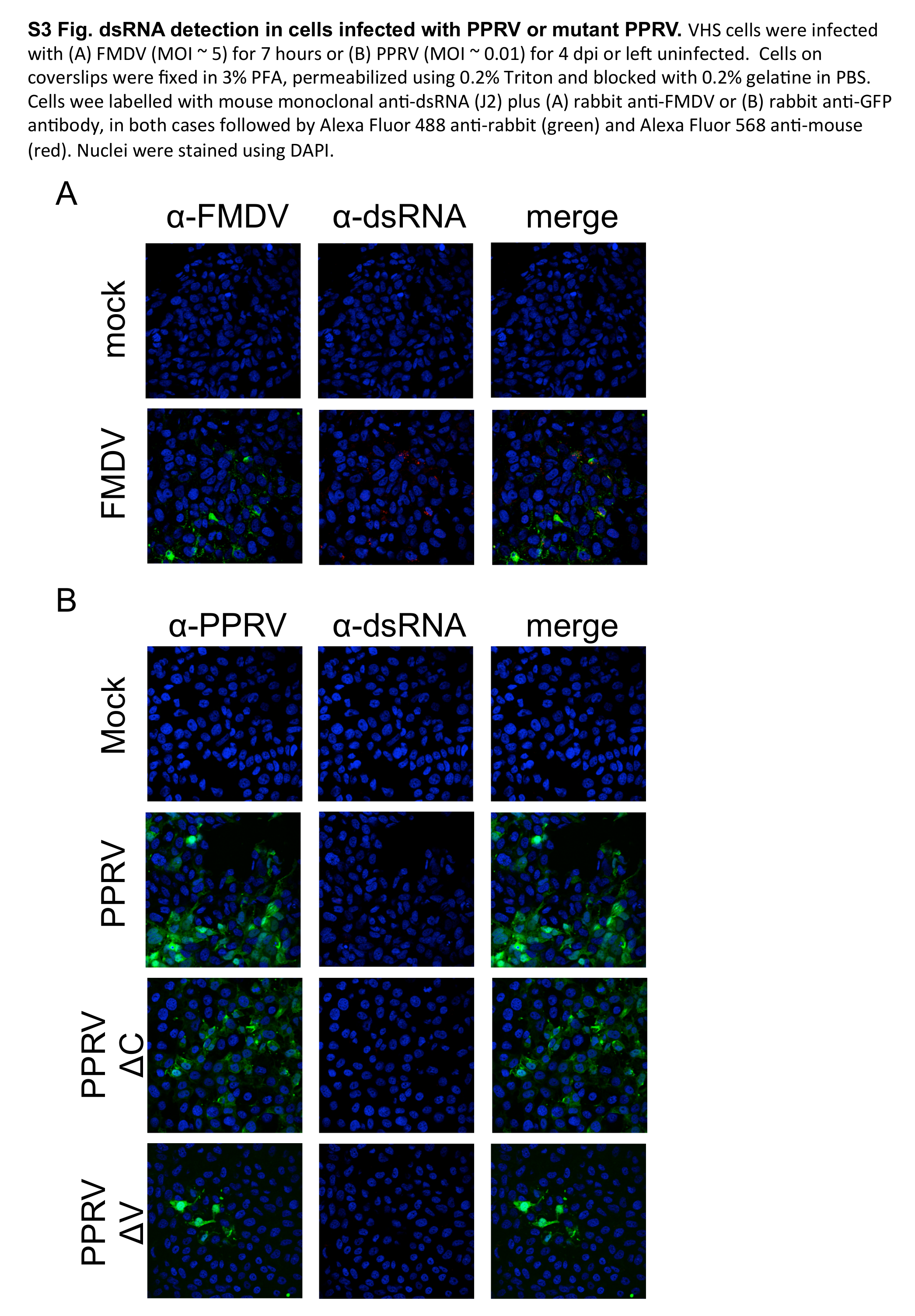

Supplement: S3 Fig — (TIFF) [file pone.0177300.s003.tiff]
